# Big Data, Big Waste? A Reflection on the Environmental Sustainability of Big Data Initiatives

**DOI:** 10.1007/s11948-019-00171-7

**Published:** 2019-12-23

**Authors:** Federica Lucivero

**Affiliations:** Ethox Centre and Wellcome Centre for Ethics and Humanities, Nuffield Department of Population Health, Big Data Institute, Li Ka Shing Centre for Health Information and Discovery, University of Oxford, Oxford, OX3 7LF UK

**Keywords:** Big Data, Environmental impacts, Materiality, Responsibility, Sustainability

## Abstract

This paper addresses a problem that has so far been neglected by scholars investigating the ethics of Big Data and policy makers: that is the ethical implications of Big Data initiatives’ environmental impact. Building on literature in environmental studies, cultural studies and Science and Technology Studies, the article draws attention to the physical presence of data, the material configuration of digital service, and the space occupied by data. It then explains how this material and situated character of data raises questions concerning the ethics of the increasingly fashionable Big Data discourses. It argues that attention should be paid to (1) the vocabulary currently used when discussing the governance of data initiatives; (2) the internal tension between current data initiatives and environmental policies; (3) issues of fair distribution. The article explains how taking into account these aspects would allow for a more responsible behaviour in the context of data storage and production.

## Introduction

In November 2014, an independent expert group appointed by the United Nations Secretary General issued a report offering recommendations on how the data revolution can be mobilised for sustainable development (United Nations (IEAG) Independent Expert Advisory Group on a Data Revolution for Sustainable Development 2014). The document shows how the increasing volume of data, passively generated and automatically collected, enables policy makers to monitor and achieve the Sustainable Development Goals (SDGs) that were announced in the 2030 Agenda (United Nations 2015b). According to this document, the “health and wellbeing” goal can be facilitated by collecting data on movements of mobile phone users to predict spread of infectious diseases; the “clean and affordable energy” goal can be achieved by reducing waste thanks to electricity, gas and water consumption data collected by smart sensors; whereas “climate action” is enabled by using satellite and other data to track deforestation. The report also highlights several challenges to sustainable and fair development raised by the current data deluge, or Big Data. The expert group encourages measures to develop standards of data quality across the global scientific community and to ensure that data are inclusive of different populations across society and around the world—“a world that counts” everyone, where no one is invisible. It also highlights that the massive acquisition of personal data, often through the passive digital footprint of people conducting their everyday life, should not jeopardise human rights, invade people’s privacy, be misused or foster inequalities because of differences in data production, access and use across populations.

Although balanced in discussing opportunities and risks of Big Data, this document and the recommended initiatives that followed up surprisingly neglect a crucial risk for sustainable development: the fact that the data revolution threatens sustainable development because of its environmental footprint. It is in fact widely acknowledged in the context of environmental research that Information and Communication Technologies (ICT), in general, and data centres and cloud computing—the backbone of Big Data—in particular, have a heavy footprint featuring high consumption of non-renewable energy, waste production and CO_2_ emissions (Pohl et al. 2019; Whitehead et al. 2014; Williams 2011). This ambiguous relationship between Big Data and sustainability is well addressed in the literature providing quantitative environmental impact assessments as well as by cultural and media studies and anthropology scholars exploring the infrastructural and material dimension of data initiatives in situated contexts (Hogan 2018; Holt and Vonderau 2015; Vonderau 2017). Despite this body of work, environmental issues are surprisingly absent, not only in policy initiatives supporting Big Data, but also in the recent literature on the ethics of Big Data.

Set in the context of growing enthusiasm for Big Data initiatives, an increasing number of scholars have examined a series of ethical concerns. These include the need to strike a balance between the use of large databases for research alongside the protection of privacy of data subjects (Craig and Ludloff 2011), the power imbalances between users of digital services and private companies (e.g. Sharon 2016), the ownership rights for data subjects (Wilbanks and Topol 2016), and the inequality between rich and low and middle-income countries in accessing data (Boyd and Crawford 2012).^1^ The ethical challenges and guidelines of the UN Expert Group report reflect a similar focus on data protection, security and justice. The problematic environmental impact of data infrastructures has not appeared so far in the analyses of the ethics of Big Data. This paper argues that an understanding of how Big Data initiatives are materially constituted and interact with the physical environment reveals ethically relevant issues that require close consideration by ethicists and policy actors.

The aim of this paper is therefore to draw scholarly attention to the ethical implications of the environmental impacts of the data revolution and to articulate the resulting individual and institutional responsibilities. To this aim, next "The Societal Value of Data” section examines how data play a crucial role in modern economies and how their production and storage is often incentivised via public and private programmes contending the social value of data initiatives. However, several studies on the material substrate of data and the impact of the physical environment show that data initiatives are far from being an endless and harmless resource (“The Weight of the Cloud and the Environment”). Drawing on these studies, in “The Ethics in the Matter” section, it is argued that such material substrate of data and its effects on the physical environment are ethically problematic for three reasons: Firstly, the implicit normativity in the vocabulary that is currently used when discussing the governance of data initiatives hides their material substrate and encourages unsustainable behaviours (of individuals and institutions). Secondly, the internal value tension between current data initiatives and environmental policies requires a thorough assessment of the social desirability of data initiatives. Thirdly, decisions are likely to be made concerning data storage that will raise issues of unfair distribution. In the last section of the paper, some policy implications of these ethically critical aspects are discussed and the role that ethicists can take in the debate is explored.

## The Societal Value of Data

In the last 10 years, industry, media and policy makers have drawn increasing attention and support to Big Data initiatives. Such growth of Big Data is driven by a number of technological advancements: the possibility of collecting new sources of data via the growing availability of wearable sensors and mobile phones, the digitation of processes and services, such as banking or medical records, and the use of sensors collecting information about the environment (Harriss 2014; Kitchin 2014; Mayer-Schönberger and Cukier 2013). At the same time, enhanced computing capabilities allow fast processing and intensive storage of data, while cloud services enable researchers to access and process large and integrated databases from different and distant locations. The potential of storing and processing large amounts of data has been seen as ground-breaking in many societal domains: for example, the collection and analysis of consumers’ preferences and spending patterns allow more targeted and effective marketing strategies, the processing of transport and travel preferences enables drivers to predict and avoid road congestion and accidents, the accumulation of information about students’ progress and habits allows for understandings of learning patterns and the design of more effective educational programmes. Health is another domain wherein data is considered crucial: the integration of primary and secondary care databases, for example, would enable the use of complete medical records, not only for care purposes, but also for biomedical research. Having this large amount of information and integrating it with data coming from other clinical (e.g. prescription data) or non-clinical sources (for example, location data or lifestyle data, such as people’s work-out habits) is expected to enable a better understanding of disease patterns and, as a result, the design of specific prevention measures (Weber et al. 2014).

In this context of increasing data collection, more elaborate processing techniques and availability of cloud services, many policy and industry documents present data as a solution to societal challenges as data are considered a resource for societal improvement and growth and a means by which to promote societal wellbeing and shared societal values (Morozov 2013). In the United Kingdom, for example, the NHS Forward View paper explicitly sets the English healthcare agenda around the importance of information and data, and encourages initiatives to enlarge or integrate existing datasets and create new ones (NHS England 2014, but also European Commission 2018 and UK Department of Digital Culture, Media and Sport 2017).

Despite the large involvement of Big Tech industry and private partners, data is presented as a tool for delivering the common good (Sharon 2018). As the European Union puts it, data foster not only “competitiveness, innovation, job creation” but also “societal progress in general”. The UN initiative—described in the Introduction of this paper—fostering the use of Big Data to achieve the sustainable development goals offers another example of this trend:

Data are the lifeblood of decision-making and the raw material for accountability. Without high-quality data providing the right information on the right things at the right time; designing, monitoring and evaluating effective policies becomes almost impossible. (United Nations (IEAG) Independent Expert Advisory Group on a Data Revolution for Sustainable Development 2014, p. 3)

According to this vision, data allow policy actors to “know more about the state of the world, and particularly the poorest people in it” (*Ibidem*, 4). They also allow for understanding of what governments need to do in order to achieve the Sustainable Development Goals, to monitor how policies and measures work towards these goals, and in this way provide transparency, which facilitates accountability for failure. In order to do this, data need to be representative of the all world population and this requires filling existing gaps in current data availability: it needs to ensure that data are collected about people, groups and regions that are currently “uncounted”. To this aim, data initiatives that gather more information on people who are left behind in the current data deluge are encouraged and funded through several programmes and public–private partnerships (see for example, https://www.unglobalpulse.org/about-new).

This data enthusiasm for the public good supports the cause for more (and better) data. Data are already copiously generated in many daily activities: by social network users, by people on their daily commute to work, by connected sensors increasingly present in machines, trains, fridges, and roads. It has been estimated that from 2005 to 2020 the digital universe will grow by a factor of 300, from 130 exabytes to 40.000 exabytes (40 trillion gigabytes) (Gantz and Reinsel 2012). With a peak of 163 zettabytes in 2025 (Reinsel et al. 2018).^2^ A recent report from International Data Corporation (IDC) forecasts that only the data generated by 41.6 billion people connected to digital “things” such as smart fridges, cars or smart speakers (IoT devices), the focus of the upcoming “5G revolution”,^3^ will generate 79.4 zettabytes (ZB) of data in 2025.^4^ As data initiatives are strongly supported and heavily funded both publicly and privately—moved by the assumption that a greater amount of data enables better analyses and improved knowledge—data production and collection is incentivised beyond its already high volume.

The growth in data production seems therefore both an enabling factor of the Big Data enthusiasm and its cause. Big Data, however, are not only an opportunity for sustainability, they also have some risks beyond the sensible preoccupation for privacy and security addressed by the Risk, Benefits and Harms tool produced by the United Nations initiative Global Pulse.^5^ High data volume requires extensive facilities for storage using natural resources such as water and non-renewable energy that require maintenance and accessibility. Furthermore, the manufacturing and disposal of devices to collect and process data emits polluting substances. The data revolution, advocated as a vehicle to achieve sustainable development, is supported by technologies that endanger sustainability and the environment by other means. Digital data are not, as some commentators have described them to critique the over-utilised analogy with oil, a “super-abundant” always available resource (Rajan 2017). It is not clean oil. Big Data has a big footprint as it usually needs non-renewable energy and limited resources to function. As discussed in the following, such implications need to be carefully considered in the discussions concerning the role of Big Data for the public good and sustainable development.

## The Weight of the Cloud and the Environment

The ambiguous relationship between sustainability and Information and Communication Technology (ICT)—on which Big Data initiatives rely—has been remarked on by scholars in environmental studies as well as computer science/engineering research for two decades. As it has been clearly put in the preamble of the recommendations signed by 200 participants in the first conference on ICT for Sustainability (ICT4S) held in Zurich in 2013:

The transformational power of ICT can be used to make our patterns of production and consumption more sustainable. However, the history of technology has shown that increased energy efficiency does not automatically contribute to sustainable development. (Hilty et al. 2013: 289)

Although in the early days, technology enthusiasts praised the positive impact of the internet on the environment by reducing gas emissions in other sectors, for example, in transportation (Romm et al. 1999; Turner et al. 2009), more critical responses to these expectations have been given by scholars in environmental and sustainability studies. Supporting this, Berkhout and Hertin (2004) argued that digital technologies should be “re-materialized” as their physical dimension and environmental impact needs to be acknowledged and assessed. Some experts in the fields of computer science, engineering and environmental science have acknowledged this internal conflict and have tried to resume it by addressing the issue of sustainability (intended as an attempt to fulfil human needs while using global resources in a way that does not deplete them and make them unusable by future generations) in relation to ICT (Hilty and Aebischer 2015). ICT have the potential for reducing environmental impacts by replacing more polluting or energy consuming products (i.e. the car to go to the grocery store, local library or public office) or making some processes more efficient (for example, through navigation systems that suggest driving routes that are less trafficked and require less driving time).^6^ However, ICT products and services also cause environmental impacts.

Environmental impacts are different in type (Berkhout and Hertin 2004; Bieser et al. 2018; Williams 2011). The more direct ones concern the “physical level, where ICT is physically embodied in an infrastructure and a set of devices” (Williams 2011: 354). A frequent measure of ICT direct environmental impact is the energy used in the operation of ICT. Increased technology performance and efficiency has brought an overall decreasing of the operational energy efficiency which is much lower in comparison to other products (such as automobile or buildings). However, the energy used during manufacturing of ICT is much higher compared to other products and draws attention on the need to assess the entire life cycle of ICT products (Williams 2011).

Since the late 1990s and across the 2000s, there has been an increasing interest by the popular press and civil society, and consequently the industry, in the direct electricity consumption of data centre facilities (Glanz 2012a, b; Markoff 2011a).^7^ Data centres are the core of the data revolution, “the central nervous system of the 21st century” (Whitehead et al. 2014: 152) as they house servers, and networking and storage equipment, enabling services such as cloud computing. They are infrastructures that offer physical place for IT equipment (computer, servers, data storage devices and routers) and support the energy intensive computing that is needed to: (1) store, manage and process digital data and (2) provide applications and services for data processing. Data centres consume an increasing quantity of energy to run their operations and cool down the servers (Avgerinou et al. 2017; Whitehead et al. 2014). They also need to run diesel generators in case of electricity shortages to keep the servers working, which produces greenhouse gas emissions with consequential impacts for climate change. According to estimates, because of advances in cloud computing and growth of the use of Internet services, data centres have the fastest growing carbon footprint from across the whole ICT sector which needs to be monitored (Avgerinou et al. 2017). In parallel to research on data-centres energy consumption, Data-Center Dynamics (DCD) started a yearly census of the industry and different metrics have been introduced (Whitehead et al. 2014). At the same time, there is still uncertainty concerning the precise values of such consumption and its future growth as projections are continuously revised and real data is difficult to acquire in a context of proprietary rights and constantly changing technologies.

The picture becomes even more varied and complicated when other factors are considered. First of all, energy consumption and greenhouse emissions of data centres are only one direct impact of ICT. The disposal of computing hardware produces harmful emissions and requires further attention. Notably, ICT disposal is particularly environmentally detrimental in informal recycling contexts in low and middle income countries (with lower environmental controls) where recovery of valuable materials in ICT hardware (for example, copper or gold), through practices like incineration, results in aggravated environmental pollution (Williams 2011). Secondly, assessing environmental impacts requires taking into account the indirect effects of ICT relating to users’ behaviours and practices (Berkhout and Hertin 2004; Bieser et al. 2018). For example, a technological practice like teleworking that is less energy consuming than driving to work may induce some energy consuming behaviours that negatively affect the environment: for instance, teleworkers may use their time saved on work commutes to drive to other places or they may spend the money saved in purchasing products with high environmental impact (Williams 2011). Thus, even if a technology’s higher performance makes it more efficient and allows for a decrease in its energy consumption and price, consumers may spend more money in ICT with a general increase of environmental effects. These are also referred to as “rebound” effects.

Indirect impacts are quite difficult to assess because they require larger behavioural and social aspects to be factored into the evaluation. Explanations of technological change at the societal level must also be linked to explanations of environmental implications (Berkhout and Hertin 2004). People’s everyday practices in using the internet, for example, the time of the day when they view videos on their mobile phone or computer, is also an important variable for data demand and therefore energy consumption. These trends are important to understand if environmental impacts (Morley et al. 2018) are to be accurately assessed. Too often, however, user related effects and rebound effects are excluded from assessments of the environmental impacts of ICT (Pohl et al. 2019). Limitations of existing methodologies in assessing all the relevant variables and tackling uncertainties call for nuanced positions and continuous monitoring of ICT implications.

In sum, it is well acknowledged that ICT in general, and data centres in particular, have an ambiguous relationship with the vision of sustainability. Despite the promises of delivering a more sustainable world, ICT also compromise this very vision through their high energy consumption and carbon footprint. Estimates of current and future environmental impacts are not definitive as they are still uncertain and incomplete, but they are nonetheless needed in order to keep track of the overall situation.

In this context, the broader political and social context needs to be considered. In 2006 and 2007, the United States Environment Protection Agency (EPA) held two public meetings that highlighted the need for data centres to become more energy efficient (Environmental Protection Agency 2007). They especially focused on putting restrictions on back up diesel generators (that are used in case of power outage) because of their air polluting emissions, but this is only one of the environmental impacts of the data revolution. Predictions of growth in data production and energy consumption have been picked up by the media and civil society who question the environmental costs that escalating energy consumption has for society (Markoff 2011b; Glanz 2012a, b). Since 2011, Greenpeace has published a series of reports that showcase which digital service providers have adopted energy saving measures and are therefore “green” companies.^8^ The list is intended to guide users of digital services to choose responsibly what services to use, on the basis of the providers’ environmental footprint.

﻿As a consequence of escalating energy costs and the questions being raised about environmental impact in the public domain, the Big Tech industry has invested in improving the efficiency of data centres for data storage and transmission. The movement of many data servers to colder climates, besides cutting energy costs by using cheaper geothermal and hydro-electricity, and by taking advantage of the colder climate that requires less electricity to cool the servers, have often been presented as solutions that address environmental concerns (Smith 2013). BigTech companies praise themselves for investing in energy efficient technologies, for example, Google announced in 2016 that the company would reach a milestone of using 100% renewable energy by 2017.^9^ Decreasing the energy costs of data farms is, of course, noteworthy, but this only addresses one type of environmental impact that related to the power needed to keep the servers running. There are, however, many other aspects to consider, such as material disposal and the rebound impacts mentioned above. Furthermore, the data economy itself drives increases in data infrastructure. While Moore’s laws of exponential decrease of computer chip transistors size has been reconsidered (Waldrop 2016), the increasing use of internet, online services (including apps and cloud services), and expectations of continuous connectivity and connected objects (the “internet of things”) nevertheless raises the demand for data and service availability, which in turn requires redundancy of data that, in order to be easily accessible, need to be stored in servers in multiple sites and centres. In this context, forecasts are that data centres will expand spatially (Carlini 2018) and even if efficiency is key, broader questions about the desirability of such a data revolution require attention.

## The Ethics in the Matter

The environmental impacts of Big Data initiatives draw attention to their material substrate and infrastructural dimensions. Such materiality is not value-free, instead it has political and social implications, as has been highlighted by scholars in the field of internet studies (Sandvig 2013), media and cultural studies (Coté 2014) and anthropology (Pink et al. 2016), who draw on social constructivist and post-human theories (like Actor-Network Theory) and human–computer interaction approaches (Leigh Star 1999). Going beyond the study of the symbolic and language-related aspects of the virtual world, these scholars turned towards an examination of the material character of the internet: they describe the material constitutions of networks (Fuller 2003; Manovich 2001), wireless connections (Mackenzie 2010) and internet infrastructures and critically explore their connections with epistemic practices or power structures (Sandvig 2013). This “material turn” in internet studies and Science and Technology Studies considers material and physical infrastructure as socially constructed, and societal relations as constructed by such infrastructures that benefit some and marginalise others. In this sense, technological choices are considered as cultural, social, political and ethical. Pioneer scholars in this field, like Susan Leigh Star (1999), have described and made apparent physical parts of the internet that would otherwise be invisible (wireless signals, buried wires, fibre optic lines)^10^ in order to uncover the tacit, intangible labour and the “politics” that are involved in practices and routines around these systems. Such practices are not only related to the design of digital services and systems, but also to their use and governance (DeNardis and Musiani 2016). Internet and media studies have therefore moved from an understanding of virtual communities as disembodied identities to a focus on the continuous relationship between online and offline activity thus challenging the virtual versus real distinction (Pink et al. 2016). In many cases, challenging the relationship between the digital and the material and acknowledging the material character of digital infrastructures, content and context is a way to question the online/offline dichotomy.

More recently, a growing body of research in cultural and media studies has also provided insight into the situatedness and material substance of data centres (Burrington 2014; Hogan and Vonderau 2019; Holt and Vonderau 2015; Hu 2015; Taylor 2017a; Vonderau 2017) Besides highlighting the environmental implications of the materially heavy infrastructure that enables the data economy, these studies discuss its political and cultural dimensions. Through discourse analyses and ethnographic studies, this research has shown how the cloud is localised within specific geographical and historical contexts with some important geopolitical consequences. For example, in her anthropological inquiry into the relocation of Facebook servers in peripheral areas of Sweden, anthropologist Asta Vonderau (2018) shows how this move has contributed to redefining the local identity of the region, creating new geographical balances where the old national peripheries become new centres of the global cloud, which has triggered conflicts between communities. Media theorist Mél Hogan has described how Big Tech promotes a narrative of environmental concern proposing themselves as the most suitable industry to manage natural resources (Hogan 2018). At the same time, however, they also generate data and fast connection demand, thus encouraging consumption and sustaining their own economy: their sustainable discourses coexist with their neoliberal practices.

The environmental implications and political and cultural dimensions of the data revolution have strong normative implications that have so far been ignored by ethicists. This is surprising because not only philosophers and ethicists of technology, together with STS scholars, have reflected on the moral implications of technical design choices of digital systems in shaping human experiences, actions and identities (see for example Achterhuis 2001; Oosterlaken and van den Hoven 2012; Verbeek 2005; Winner 1999), also, the ethical reflection on implications of Big Data has boomed in the last 10 years (Mittelstadt and Floridi 2015). Despite this relevant scholarly work, the topic of the environmental implications of Big Data and their material infrastructure has gone largely unremarked.

The paper now discusses three ways in which the materiality of data has ethically relevant implications and would therefore benefit from research in this field. Firstly, it explains the implicit normativity in the vocabulary that is currently used when discussing the governance of data initiatives, and how this normatively laden vocabulary influences understandings of responsible behaviour. Secondly, it explores the internal tensions between current data initiatives and environmental policies and points out the need for a thorough assessment of benefits and risks. Thirdly, it analyses issues of fair distribution in the context of decision-making on data storage practices. For each of these aspects it will be pointed out why these issues should matter to ethicists, how they fall within their interests and how ethicists could contribute to the debate. The last section of the paper further reflects on the role of the ethicist and some of the policy implications for these aspects.

### The Ethics in the Metaphor: From Language to Actions

Media and lay discourses often suggest that the digital is somewhat immaterial. As pointed out by several commentators, using the term “cloud” to refer to computing and internet networks is a misleading metaphor (Holt and Vonderau 2015; Hu 2015; Taylor 2017a). It suggests something impalpable, fluffy, untouchable, light, and transparent. This language strategically obscures the materiality of the infrastructure as well as its geographical presence and environmental impact. Cloud computing is in fact a highly tangible and touchable assemblage of material and heavy stuff. The material substrate of the cloud is made of cables, wires, servers, and shelves in buildings in every corner of the globe. Similarly, the language used to refer to data as an “unlimited and superabundant resource” implicitly suggests that data are virtual goods, always present, an ever increasing and never-ending resource, in contrast to other resources (such as oil, water, or land) that are limited in quantity and need careful management. However, how data relies on limited resources in order to be stored and processed suggests that it is not superabundant.

The use of this type of language in relation to the storage of data induces false assumptions as it tends to hide what the “material turn” in internet and media studies has highlighted: that there is a continuity of offline and online life. The digital has material implications for the physical “real” world, but the language used obscures this. Places, infrastructures, and buildings all play a role in constituting the online world, and vice versa: online behaviour also has material implications in the physical “real” world. False assumptions, created by misleading language, influence people’s understanding of the data universe and economy, and shapes their attitudes towards it. For example, it suggests that the virtual environment is drastically different from the material, physical environment in which we live our offline lives, where we eat, sleep, watch movies, have sex, and throw away our garbage. However, digital behaviour interferes with the physical environment and not always in a positive way. Buying or selling bitcoins, storing large amounts of photos on cloud services, binge watching TV are all online activities, but their implications are not relegated solely to the digital environment as they also require material and limited resources (space, water, electricity, and fuel) and therefore have serious implications for the natural environment.

Metaphors and language suggesting otherwise are culpable of obscuring the physical character of data and its implications for the offline world. Data consumption is no less environmentally problematic than material goods consumption; and the paperless, computer and data intensive office is not an ultimate solution to environmental issues, but instead creates new ones [see also Tenner (1996)]. Highlighting the material consequences of online behaviours and their impacts on the environment is crucial in order to draw attention to individual and institutional responsibilities in terms of the common good. This is even more relevant in societies where we acknowledge the importance of protecting the environment by incentivising “green” activities aimed at reducing individual and collective carbon footprints. Production and storage of data can be environmentally problematic and, as such, the sustainability of digitally-rich activities and behaviours should be part of the conversation on Big Data.

Besides hiding responsibilities, the language referring to data as an “unlimited and superabundant resource”, which only needs to be collected and managed in order to be used in a meaningful and socially beneficial way, implicitly calls for incentivising policies and activities that promote data initiatives. The risk is that the logic of such language lends governors and policy makers to think that by promoting numerous data initiatives they are always promoting the public good. Again, however, the environmental costs of these implications should be considered when assessing how, or whether, these initiatives foster the public good.

According to a pragmatist view on ethics (Keulartz et al. 2002; Lucivero 2016), ensuring that the normative assumptions in industry and policy discourses questioned are disclosed, falls into the remits of ethics. Vocabularies have a moral character as they justify some actions and forbid others as well as contributing to knowledge and distributing responsibilities. Reconsidering the validity of such metaphors and using a vocabulary that highlights the material and physical character of data storage could help in enhancing awareness of the consequences of some actions and allow space for discussing individual and institutional responsibilities towards data handling, storing and production. Ethicists can have a role in facilitating such discussion as a first step in questioning the presumed benefits of data initiatives.

### The Ethics in the Visions: Evaluating Data Initiatives

The second morally relevant consideration behind the material nature and environmental impact of Big Data is that such impacts should be considered when assessing the overall value of these initiatives; this is important in relation to the societal value of sustainability that, according to UN and industry visions, Big Data can promote. With increasing understandings of the relationship between human intervention and the natural environment, public health authorities and governments have placed much emphasis on the need to reduce anthropogenic changes to the environment that can have negative impacts on human health. Take for example the UN 2030 Agenda for Sustainable Development that was adopted in 2015 by 193 countries (United Nations 2015a).^11^ Nine of the 17 goals explicitly refer to the environment and sustainability as a value: not only as goals clearly referring to policies actively promoting environmental goods^12^ but also as goals linking innovation and economy with the overarching aim of sustainability. These include, for example, ensuring access to affordable, reliable, sustainable and modern energy (goal 7) the development of clean industry and environmentally sound technologies and industrial processes (goal 9) or the sustainable management and use of material resources (12.2); the reduction of waste generation through prevention, reduction, recycling and reuse (12.5) and the encouragement of companies to adopt sustainable practices and integrate sustainability information into their reporting cycles (12/6); the importance of raising awareness (12.8), and the importance of taxing inefficient and unsustainable practices (12.c). Sustainable development is strictly linked to the improvement (or non-deterioration) of the environmental conditions in which people live and to the protection of the ecosystem.

Goals and values, such as sustainability, therefore, clearly permeate the visions of policy makers and broader society and are used to justify policy decisions. They are guiding normative visions for policy makers and broader communities as they propose more desirable futures (Grin and Grunwald 2000; Jasanoff and Kim 2013). Policy visions of Big Data for sustainable development (or health) can be considered as sociotechnical imaginaries with a performative role. In the world of the EU, data are “*an essential resource*, *for economic growth, competitiveness, innovation, job creation and societal progress in general”*. Data initiatives are promoted within the context of modern economies and are expected to have great benefits for populations and individuals. The normative dimension of such visions emerges in the fact that they depict images of futures that are presented as desirable for societies and individuals (for example, healthier and more sustainable) and suggest ways to realise them. This normative dimension suggests that data are good, not only for individuals and private actors, but also for societies at large. However, data initiatives can also have negative environmental impacts. Therefore, on closer examination, the guiding values of data economy policies seem to be at odds with the guiding values of sustainable development predicated in the same policies.

Visions of Big Data *for* sustainable development (fighting, for example climate change) collide with the reality of Big Data, which can work *against* sustainable living. The two visions are not necessarily incompatible and can be considered as two sides of the data revolution coin: as we have seen in the previous section, ICT and sustainability have an ambiguous relationship and yet there are ways for ICT to contribute to sustainability. To further complicate the matter, sustainability itself is a “contested value” as it is conceptualised in different and sometimes conflicting ways to normative underpinnings (Van de Poel 2017). For these reasons, one cannot expect complete consistency in policy visions of Big Data for sustainable development. At the same time, it is important to make policy makers, funders and institutions aware of the ambiguity of the relationship between sustainability and Big Data in order to nuance expectations and initiate a dialogue about underlying values, priorities and trade-offs. Ethicists can contribute to more responsible public policy by raising awareness of these ambiguities, fostering a weighing of benefits and drawbacks of data initiatives, uncovering hidden value conflicts and making governing bodies and public institutions more critical of the rhetoric of data optimism strategically advanced by Big Tech. For example, Big Data projects could be made accountable for assessing their environmental impacts, in the same way that they are required to account for the way they tackle issues of privacy and security. Documents such as the United Nations report on Big Data for Sustainable Development discussed above, do not list environmental issues amongst the challenges of Big Data initiatives as they focus primarily on issues of privacy, security and fair representation and access (United Nations (IEAG) Independent Expert Advisory Group on a Data Revolution for Sustainable Development 2014).

As explained in “The Weight of the Cloud and the Environment” section, environmental harm is still uncertain and difficult to measure. This uncertainty suggests that current measures of environmental impact assessment may fall short when asked to provide a definitive answer or a clear-cut weighing of benefits and drawbacks. This complexity demonstrates the need for an ethical and philosophical reflection on impacts that takes account, not only quantitative aspects, but also of qualitative, behavioural and moral dimensions.

Understanding how data initiatives mobilise different values and discussing how to balance them is therefore crucial to a sound ethical discussion. Ethicists can also help in the assessment of the broader benefits and drawbacks of data initiatives for health. Such assessments are not a one-off, preliminary, “measuring” exercise (wherein impacts are quantified and weighted before specific data initiatives are given a green light) but require a continuous reflection that works in parallel with data initiatives and which also focuses on qualitative impacts.

### The Ethics in Decision-Making: Allocating Resources and Changing Landscapes

A third ethically problematic aspect related to the material nature of data relates to the criteria that drive decision-making processes around allocation of data-relevant resources. Data storage and use depend on the availability of land and resources such as water and fuel. Since these resources are scarce, we cannot exploit them limitlessly. Technical and logistical solutions to use less of these resources are a way of dealing with this problem: these solutions include, for example, the establishment of data centres in Nordic countries where less energy is needed to cool down servers.^13^ However, these solutions may create new problems: for example, the geographical location of data centres raises questions of legitimacy and autonomy concerning decisions over the landscape and use of the natural environment. Data infrastructures not only alter the physical space, but also the socio-economic organisation within that space and beyond. Data centres, for example, are currently modifying natural and urban landscapes with their distinctive architectures (Burrington 2014; Taylor 2018). Vonderau shows that in placing data centres in the peripheric North, aspirations of local change face the reality of limited jobs offered to local communities in a highly automated industry (Vonderau 2018). These geopolitical revolutions in specific areas raise questions of how these investments benefit local residents, how the new arrangements change social and economic configurations both at the local and the national level and who gains and who loses in these changing interactions. For example, in the case of digital device disposal discussed above, the location of disposal facilities and informal recycling centres in low- and middle-income countries raises questions concerning the fair distribution of benefits and damages of the digital revolution, where more vulnerable populations suffer the environmental consequences of rich countries’ increasing digital demand. Furthermore, it is legitimate to question who should be involved in such decisions regarding location and management of such infrastructures.

Proximity to data centres and geographical distribution of data centres raise questions of fairness in the context of a growing data economy. For example, it has been highlighted that the proximity or distance from relevant infrastructures determines conditions of access to services. In 2013, architecture reporter Andrew Blum, published the book *Tubes*: *Behind the Scenes at the Internet* where he describes 2 years of research in which he attempted to better understand the materiality of the internet by visiting the real places on the map where the internet is built, provided, connected and distributed (Blum 2013). In describing the physical details, buildings, people and infrastructures that make the network a real and tangible thing, capable of modifying geographies and the landscape, Blum challenges the metaphors of the digital as a space beyond place. Not only does he show that the internet is a place but also that data is located in some places more than others. The proximity to data servers allows information to travel at a faster speed, which is quite relevant for certain businesses. The fact that the old telecom company Verizon has recently sold a building in central Manhattan to a real estate agent for data companies, highlights the increasing need for data companies to locate in urban areas, such as central Manhattan, in order to allow stock market companies to work at the speeds necessary for their business (Carp 2013). As the physical location of infrastructures determines speed of access to services, decisions concerning the location of centres cannot ever be neutral, a matter which has societal implications. So, if proximity to data infrastructures has competitive implications for the fast accessing or processing of data, who will decide the activities that require more (and more proximity to) data storage and processing facilities? Furthermore, what criteria would drive decisions on whether priority should be given to healthcare databases, stock market programmes, or social media and consumer storing servers? The location of data centres will also have implications for who has faster access. This is increasingly important in the context of a data economy where access to data means access to wealth and economic development, as this implies that the choice of place will also have an impact on local economies. Given that data centres are increasingly being moved to cold countries, as warmer climates are not suitable for their placement, how can the growth of economic gaps between warm countries and cold countries be avoided? Leaving these decisions in the hands of the commercial sector could be problematic as issues concerning the common good and justice may be overlooked in favour of the economic gain of a few private actors.

Another set of questions related to fairness in deliberations in contexts of scarcity concerns the criteria to decide what can be stored and what needs to be deleted. In a context where digital material occupies physical space^14^ and cannot be endlessly kept, decisions need to be made with respect to what information or knowledge should be kept or deleted. Interestingly, Jonathan Mirand and colleagues have explored these issues in the project “The Archive Documentary”^15^ where they highlight how, as digital space needs to be decluttered, some digital production will inevitably need to be erased to make space for more. Their project aims at creating an archive that saves artistic and cultural productions as often these are considered less appealing for industry and, as such, they become lost. More generally, this artistic project reminds us that when decisions concerning the placement of data centres or the storing of data are involved, criteria that justify the legitimacy of these decisions need to be explored. Who decides what is important and what the criteria are for determining the value of data?

These sets of questions show that data initiatives do not build on superabundant resources and although data can ideally be produced in unlimited ways, the resources that are used for their storage and processing (e.g. water, physical space, electricity) are finite. In this context of scarcity, decisions of resource allocation need to be made. What is up for discussion is: how the data revolution distributes environmental benefits and losses among groups and communities, what type of inequalities it creates and who is responsible for them, whom should be involved in the decisions about distribution of scarce resources and environmental outcomes, what criteria will need to be considered (e.g. not only economic and efficiency-based criteria, but also those based on fairness) and what values should guide these choices (e.g. economic utility, cultural value). These are questions of social justice and as such would be enriched by an ethical analysis. Political theorists and environmental activists have been raising similar questions under the banner of “environmental justice” (Bullard 2005; Schlosberg 2009, 2013). In these discussions, the distributive issues around environmental benefits and disbenefits have been explored, but also the structural reasons for such imbalances as well as issues of participatory parity. As the digital revolution creates new inequalities that have motivated the need for addressing issues of “data justice” (Dencik et al. 2019; Taylor 2017b), so new issues of environmental justice arise, as not everyone “enjoys the same degree of protection from the environmental and health hazards” (US EPA, n.d.). Moreover, not everyone has equal access to the decision-making process involving data infrastructure. It is at this intersection between data justice and environmental justice—where the populations that currently suffer more from the environmental implications of ICT are also the ones that are at the present time less likely to benefit from the digital revolution—that more work needs to be done.

## Policy Implications and the Role of the Ethicist

On several occasions the new data economy has been compared to the “old” oil-focused economy. As an editorial in *The Economist* in May 2017 suggestively put it (rehashing an analogy that had been already used by several speakers in the policy, industry and academic environment): Data is the oil of the digital era (“The World’s Most Valuable Resource” 2017). In the same way that oil was a driver for change in the last century, so is data in this century. As the paper has highlighted, like oil, data production, collection and processing have an environmental impact and should not been considered as a “super-abundant” supply as suggested in some mainstream discourses. The growing data economy with its related investments and infrastructures encourages a limitless access to, and use of, data and dominates government policies and industry discourses. However, investing in the data economy, enabling access and creating demand with no consideration for the environmental impact and energy consumption is likely going to become a problem if not addressed in due time.

This paper has argued that, although Big Data initiatives can be a resource for modern and sustainable economies, the material substrate of these initiatives and their environmental impacts also raise some challenges for sustainability. These challenges have so far been neglected by ethicists. This paper, therefore, explains why this is a moral problem and how it falls into the remits of ethical analysis. Three aspects have been analysed: The first aspect concerns implicit normativity in the current language used in discussions of the governance of data initiatives and how this works to obscure individual and institutional responsibilities. The second aspect concerns the internal value tensions between current data initiatives and environmental policies and the need to assess the benefits of data initiatives in a comprehensive way. The third aspect refers to issues of social justice that are likely to emerge in a context where data storage has to be rationed and suggests the importance of democratising the discussion on distributive criteria. This paper is a call for ethicists to engage with these topics and play an active role in data policy formation.

Ethicists are particularly suited to improving mainstream expectations and policies around data and sustainability. A consequentialist focus on harms is limited because of the multiple variables involved in assessing the environmental impact of data initiatives, a pragmatist approach focusing on articulating the plurality of concrete values, situating them and considering plausible trade-offs may be more promising. More specifically, pragmatist ethicists are well suited for disentangling values and normative structures in vocabularies and discourses around data, thereby, clarifying their moral grammar and articulating value inconsistencies as well as exploring stakeholders’ normative positions and assessing how their values are represented.^16^

Ethicists can therefore play a role in mapping the moral landscape and the conflicting values implicit in the visions of the sustainability of the data revolution, and in developing criteria to assess initiatives, exploring issues of environmental justice in this context, facilitating dialogue and the participation of more vulnerable and less represented stakeholders, bringing these issues into the policy agenda and articulating necessary trade-offs. Promoting a sustainable data economy requires, not only the reduction of energy costs of data centres, but also the production of less “waste” or less data that require resources in order for them to be processed and stored (and eventually destroyed). This has three types of normative implications. The first is that we need to change discourses around data to highlight individual, commercial and institutional responsibilities; the second is that an assessment of environmental implications has to accompany a review of the opportunities of data initiatives; and the third is that we should carefully consider criteria in making just decisions around data initiatives. In the following, these implications will be discussed.

First of all, a sustainable and responsible discourse around data should be incentivised. The way in which institutions, media and industry speak of data as an immaterial and unlimited resource, the lack of recognition in metaphors like “the cloud” or adjectives like “superabundant”, conceal the material dimensions and environmental factors of data initiatives and data storing behaviour. Changing discourses enables an acknowledgement that data are a limited resource and suggests that some norms and principles that guide sustainable and responsible behaviour in the physical environment should also apply in the context of a digital environment. More practically, awareness campaigns to change people’s behaviour towards data storage could be helpful in this context: campaigns to raise awareness of the pollution of data centres, apps, computing services (see for example the annual reports “Clicking clean” published by Greenpeace: http://www.greenpeace.org/usa/global-warming/click-clean/). This is similar to what is already being done to mitigate climate change where policy makers encourage behavioural change solutions, for example, those incentivising individuals to reduce the emissions per capita or reducing their consumption profile by changing some lifestyle choices, for example, by reducing food waste, changing mobility habits (van de Ven et al. 2017). Storing data in a cloud system may be one of those lifestyle choices that people may need to consider with respect to their environmental impact.

These campaigns should therefore be seen as an institutional effort to change the dominant culture around data production and storage. This will bring about more responsibilities for institutions that will need to make more environmentally friendly options viable and affordable for citizens. In this context, institutions also have a responsibility to initiate discussions with industry and require them to develop more sustainable solutions. The US Environment Protection Agency worked towards this in 2006–2007 when data centres were instructed to be more energy efficient. Efforts in this direction should be implemented in order to ensure that the data industry addresses environmental concerns promptly.

Secondly, environmental implications should be taken as seriously as Big Data opportunities. After all, the same duties and rights that apply to the physical environment also apply to the digital environment since the digital environment is also physical. Currently, assessments of environmental impacts of data centres’ activities are based on single metrics, such as energy consumption, which do not offer a broader and holistic view of their sustainability (Whitehead et al. 2014). A reflection on the relevant parameters needs to included, encompassing not only the quantitative but also, the qualitative impacts of data centres on environment and material landscapes.

Finally, public institutions also have a role in encouraging a more general discussion on the criteria used to define what is relevant to store and what is not as well as on accountability and fairness in such decision making. This should not be left to industry to decide as this is a matter that affects entire societies and local communities. If industry relies on public support for data initiatives, it should also involve public institutions in the establishment of data selection criteria. A selective mentality should drive policy and funding initiatives and go beyond short-term processes, such as collecting data without planning how we intend to use them. Instead, questions concerning what is relevant and why, should be asked at each stage of the process in order to ensure that we are not collecting huge amounts of unnecessary data.

In conclusion, the fact that Big Data affects the environment does not imply that we should ban or discourage data intensive research or initiatives. Indeed, many human activities adversely affect the environment and yet they continue. The activity of car driving, for example, is a case in point. At the same time, however, we must try to raise awareness and promote changes that minimise the effect these activities have on the environment, for example, through incentives such as car-pooling, or policies that promote suitable alternatives, such as electric cars. This paper has argued that financially incentivising more environmentally friendly solutions to data collection and storage whilst financially disincentivising more polluting solutions (e.g. CO_2_ quota) will be essential to achieve the same goals. To reduce the adverse effects of data storage on the environment, more care must be taken in assessing data initiatives and in examining the ethical implications that arise from their material substrate.

## Footnotes

1 For a broad and exhaustive review of ethical themes of Big Data in the biomedical fields see Mittelstadt and Floridi (2015).

2 According to the International Data Corporation (IDC), 163 ZB is equal to “watching the entire Netflix catalogue 489 million times 40 trillion DVDs which would reach to the moon and back over 100 million times” https://www.seagate.com/files/www-content/our-story/trends/files/data-age-2025-infographic-2017.pdf (Accessed on 19/07/19).

3 https://worldin2019.economist.com/transformbusinessesandtheworld (Accessed on 19/07/19).

4 https://www.idc.com/getdoc.jsp?containerId=US45066919 (Accessed on 19/07/19).

5 https://www.unglobalpulse.org/privacy/tools (Accessed on 19/07/19).

6 This relationship can be distinguished between: “Sustainability in ICT” which means “making ICT goods and services more sustainable over their whole life cycle, mainly by reducing the energy and material flows they invoke” and “Sustainability by ICT” that is “creating, enabling, and encouraging sustainable patterns of production and consumption by means of ICT” (Hilty and Aebischer 2015, p. 21).

7 See also http://www.greenpeace.org/usa/global-warming/click-clean/.

8 See http://www.greenpeace.org/usa/global-warming/click-clean/. Accessed on 25/05/2018.

9 See https://environment.google/projects/announcement-100/.

10 Beyond bricks, mortar, pipes or wires, infrastructure also encompasses more abstract entities, such as protocols (human and computer), standards, and memory [as well as] digital facilities and services […such as] computational services, help desks, and data repositories to name a few (Bowker et al. 2010, p. 97).

11 See http://www.un.org/sustainabledevelopment/sustainable-development-goals/. Accessed on 25/05/18.

12 e.g. “Take urgent action to combat climate change and its impacts” (13), “Conserve and sustainably use the oceans, seas and marine resources for sustainable development” (14), “Protect, restore and promote sustainable use of terrestrial ecosystems, sustainably manage forests, combat desertification, and halt and reverse land degradation and halt biodiversity loss” (15).

13 Another solution recently explored by Google is to establish data centres floating off coast (Carroll Rory. Google’s worst-kept secret: floating data centres off US coasts, The Guardian [Internet]. The Guardian. 2013 [cited 2018 May 25]. Available from: https://www.theguardian.com/technology/2013/oct/30/google-secret-floating-data-centers-california-maine.

14 Although very often Moore’s law is quoted to justify that computing capabilities need half of the space every 2 years, it has been shown that as we approach the atomic size, such observation presents several limitations (see for example, Waldrop 2016).

15 See http://bigbangdata.cccb.org/en/tag/the-internet-archive-en/; https://archive.org/details/archive_documentary_internet_archive_sequence. Accessed on 25/05/2018.

16 For an account of the role of pragmatism in environmental ethics see (Katz and Light 2003).

## References

[CR1] Achterhuis H (2001). American philosophy of technology: The empirical turn.

[CR2] Avgerinou M, Bertoldi P, Castellazzi L, Avgerinou M, Bertoldi P, Castellazzi L (2017). Trends in data centre energy consumption under the European code of conduct for data centre energy efficiency. Energies.

[CR3] Berkhout F, Hertin J (2004). De-materialising and re-materialising: Digital technologies and the environment. Futures.

[CR4] Bieser J, Hilty L, Bieser JCT, Hilty LM (2018). Assessing indirect environmental effects of information and communication technology (ICT): A systematic literature review. Sustainability.

[CR5] Blum A (2013). Tubes.

[CR6] Bowker GC, Baker K, Millerand F, Ribes D, Hunsinger J, Klastrup L, Allen M (2009). Toward information infrastructure studies: ways of knowing in a networked environment. International handbook of internet research.

[CR7] Boyd D, Crawford K (2012). Critical questions for big data. Information, Communication & Society.

[CR8] Bullard RD (2005). The quest for environmental justice: Human rights and the politics of pollution.

[CR9] Burrington, I. (2014). The cloud is not the territory. http://creativetimereports.org/2014/05/20/ingrid-burrington-the-cloud-is-not-the-territory-wnv/. Retrieved July 13, 2019.

[CR10] Carlini, S. (2018). Data center predictions for 2019—Data centre dynamics. https://www.datacenterdynamics.com/opinions/data-center-predictions-2019/. Retrieved July 19, 2019.

[CR11] Carp, A. (2013). Inside Manhattan’s tower of internet—The awl. https://www.theawl.com/2013/03/inside-manhattans-tower-of-internet/. Retrieved May 25, 2018.

[CR12] Coté M (2014). Data motility: The materiality of big social data. Cultural Studies Review.

[CR13] Craig T, Ludloff M (2011). Privacy and big data.

[CR14] DeNardis L, Musiani F (2016). Governance by infrastructure. The Turn to Infrastructure in Internet Governance.

[CR15] Dencik L, Hintz A, Redden J, Treré E (2019). Exploring data justice: Conceptions, applications and directions. Information Communication and Society.

[CR16] Environmental Protection Agency. (2007). *Report to congress on server and data center energy efficiency executive summary*. Retrieved from https://www.energystar.gov/ia/partners/prod_development/downloads/EPA_Report_Exec_Summary_Final.pdf.

[CR17] European Commission. (2018). Communication from the Commission to the European Parliament, the Council, the European Economic and Social Committee and the Committee of the Regions on enabling the digital transformation of health and care in the Digital Single Market; empowering citizens and building a healthier society. COM/2018/233 final. Retrieved December, 2019, from https://eur-lex.europa.eu/legal-content/EN/TXT/?uri=COM%3A2018%3A233%3AFIN.

[CR18] Fuller M (2003). Behind the blip: Essays on the culture of software.

[CR19] Gantz, J., & Reinsel, D. (2012). The digital universe in 2020: Big data, bigger digital shadows, and biggest growth in the far east. IDC iView. Retrieved December, 2019, on https://www.emc.com/leadership/digital-universe/2012iview/index.htm.

[CR20] Glanz, J. (2012a). Data centers in Rural Washington state gobble power—The New York Times. https://www.nytimes.com/2012/09/24/technology/data-centers-in-rural-washington-state-gobble-power.html?ref=us&_r=0. Retrieved May 25, 2018.

[CR21] Glanz, J. (2012b). Data centers waste vast amounts of energy, belying industry image—The New York Times. https://www.nytimes.com/2012/09/23/technology/data-centers-waste-vast-amounts-of-energy-belying-industry-image.html?_r=0. Retrieved May 25, 2018.

[CR22] Grin J, Grunwald A (2000). Vision assessment: Shaping technology in 21st century society.

[CR23] Harriss, L. (2014). Big data: An overview. *POSTnotes*, *POST*-*PN*-*46*.

[CR24] Hilty LM, Aebischer B (2015). ICT innovations for sustainability.

[CR25] Hilty, L. M., Aebischer, B., Andersson, G., & Lohmann, W. (2013). *ICT4S 2013*. 10.3929/ETHZ-A-007337628.

[CR26] Hogan M (2018). Big data ecologies. Ephemera: Theory & Politics in Organisation.

[CR27] Hogan, M., & Vonderau, A. (2019). The nature of data centers. *Culture Machine*, *18*. Retrieved July, 2019, from www.culturemachine.net.

[CR28] Holt J, Vonderau P, Parks L, Starosielski N (2015). Where the internet lives. Signal traffic.

[CR29] Hu T-H (2015). A prehistory of the cloud.

[CR30] Jasanoff S, Kim S-H (2013). Sociotechnical imaginaries and national energy policies. Science as Culture.

[CR31] Katz, E., & Light, A. (2003). Environmental pragmatism. Retrieved July, 2019, from http://public.ebookcentral.proquest.com/choice/publicfullrecord.aspx?p=1144513.

[CR32] Keulartz J, Schermer M, Korthals M, Swierstra T (2002). Pragmatist ethics for a technological culture.

[CR33] Kitchin R (2014). Big data, new epistemologies and paradigm shifts. Big Data & Society.

[CR34] Leigh Star S (1999). The ethnography of infrastructure. American Behavioral Scientist.

[CR35] Lucivero F (2016). Ethical assessments of emerging technologies.

[CR36] Mackenzie A (2010). Wirelessness: Radical empiricism in network cultures.

[CR37] Manovich L (2001). The language of new media.

[CR38] Markoff, J. (2011a). Data Centers using less power than forecast, report says—The New York Times. https://www.nytimes.com/2011/08/01/technology/data-centers-using-less-power-than-forecast-report-says.html. Retrieved May 25, 2018.

[CR39] Markoff, J. (2011b). Google lobbies Nevada to allow self-driving cars. *The New York Times*. Retrieved July, 2019, from http://www.nytimes.com/2011/05/11/science/11drive.html?_r=0.

[CR40] Mayer-Schönberger V, Cukier K (2013). Big data: A revolution that will transform how we live, work and think.

[CR41] Mittelstadt BD, Floridi L (2015). The ethics of big data: Current and foreseeable issues in biomedical contexts. Science and Engineering Ethics.

[CR42] Morley J, Widdicks K, Hazas M (2018). Digitalisation, energy and data demand: The impact of Internet traffic on overall and peak electricity consumption. Energy Research & Social Science.

[CR43] Morozov E (2013). To save everything, click here: Technology, solutionism, and the urge to fix problems that don’t exist.

[CR44] NHS England. (2014). *Five year forward view*. Retrieved July, 2019, from https://www.england.nhs.uk/wp-content/uploads/2014/10/5yfv-web.pdf.

[CR45] Oosterlaken I, van den Hoven J (2012). The capability approach, technology and design.

[CR46] Pink S, Ardèvol E, Lanzeni D (2016). Digital materialities: Design and anthropology.

[CR47] Pohl J, Hilty LM, Finkbeiner M (2019). How LCA contributes to the environmental assessment of higher order effects of ICT application: A review of different approaches. Journal of Cleaner Production.

[CR48] Rajan, A. (2017). Data is not the new oil. https://www.bbc.com/news/entertainment-arts-41559076. Retrieved May, 2018.

[CR49] Reinsel, D., Gantz, J., & Rydning, J. (2018). Data age 2025: The digitization of the world from edge to core. Int Data Corp [Internet]. https://www.seagate.com/files/www-content/our-story/trends/files/idc-seagate-dataage-whitepaper.pdf.

[CR50] Romm, J. J., Rosenfeld, A. H., & Herrmann, S. (1999). The internet economy and global warming. A scenario of the impact of E-commerce on energy and the environment [Internet]. Center for Energy and Climate Solutions. https://p2infohouse.org/ref/04/03784/0378401.pdf.

[CR51] Sandvig, C. (2013). The internet as infrastructure. In W. H. Dutton (Ed.), (Vol. 1). Oxford University Press. 10.1093/oxfordhb/9780199589074.013.0005.

[CR52] Schlosberg D (2009). Defining environmental justice: Theories, movements, and nature.

[CR53] Schlosberg D (2013). Theorising environmental justice: The expanding sphere of a discourse. Environmental Politics.

[CR54] Sharon T (2016). The googlization of health research: From disruptive innovation to disruptive ethics. Personalized Medicine.

[CR55] Sharon T (2018). When digital health meets digital capitalism, how many common goods are at stake?. Big Data and Society.

[CR56] Smith, S. (2013). Arctic steampunk: The new age of cold weather data infrastructure—Thesigers. http://thesigers.com/analysis/2013/3/8/arctic-steampunk-the-new-age-of-cold-weather-data-infrastruc.html. Retrieved May 25, 2018.

[CR57] Taylor A (2017). The technoaesthetics of data centre’ White Space. Imaginations.

[CR58] Taylor, A. (2018). Failover architectures: The infrastructural excess of the data centre industry—Failed architecture. *Failed Architecture*. Retrieved July, 2019, from https://failedarchitecture.com/failover-architectures-the-infrastructural-excess-of-the-data-centre-industry/.

[CR59] Taylor L (2017). What is data justice? The case for connecting digital rights and freedoms globally. Big Data & Society.

[CR60] Tenner E (1996). Why things bite back: Technology and the revenge of unintended consequences.

[CR61] The World’s Most Valuable Resource. (2017). *The economist*. Retrieved July, 2019, from https://www.economist.com/leaders/2017/05/06/the-worlds-most-valuable-resource-is-no-longer-oil-but-data.

[CR62] Turner, V., Bigliani, R., & Ingle, C. (2009). Reducing greenhouse gases through intense use of information and communication technology [Internet]. http://download.intel.com/pressroom/archive/reference/IDCWP31R.pdf.

[CR63] UK Department of Digital Culture, Media and Sport. (2017). Data - unlocking the power of data in the UK economy and improving public confidence in its use [Internet]. Retrieved December, 2019, from https://www.gov.uk/government/publications/uk-digital-strategy/7-data-unlocking-the-power-of-data-in-the-uk-economy-and-improving-public-confidence-in-its-use.

[CR64] United Nations. (2015a). *Resolution adopted by the General Assembly on 25 September 2015 70/1. Transforming our world: The 2030 agenda for sustainable development*. Retrieved July, 2019, from http://www.un.org/en/development/desa/population/migration/generalassembly/docs/globalcompact/A_RES_70_1_E.pdf.

[CR65] United Nations. (2015b). *Transforming our world: The 2030 agenda for sustainable development* (working papers). eSocialSciences. Retrieved July, 2019, from https://econpapers.repec.org/RePEc:ess:wpaper:id:7559.

[CR66] United Nations (IEAG) Independent Expert Advisory Group on a Data Revolution for Sustainable Development. (2014). *A world that counts: Mobilising the data revolution for sustainable development*. Retrieved July, 2019, from www.undatarevolution.org.

[CR67] US EPA. (n.d.). Environmental Justice | US Environmental Protection Agency. https://www.epa.gov/environmentaljustice. Retrieved November 19, 2019.

[CR68] Van de Poel I, Kaplan DM (2017). Design for sustainability. Philosophy, technology, and the environment.

[CR69] van de Ven D-J, González-Eguino M, Arto I (2017). The potential of behavioural change for climate change mitigation: A case study for the European Union. Mitigation and Adaptation Strategies for Global Change.

[CR70] Verbeek P (2005). What things do: Philosophical reflections on technology, agency, and design.

[CR71] Vonderau A (2017). Technologies of imagination: Locating the Cloud in Sweden’s North. Imaginations Journal of Cross-Cultural Image Studies/Revue d Études Interculturelle de l Image.

[CR72] Vonderau A (2018). Scaling the cloud: Making state and infrastructure in Sweden. Ethnos Journal of Anthropology.

[CR73] Waldrop MM (2016). The chips are down for Moore’s law. Nature.

[CR74] Weber GM, Mandl KD, Kohane IS (2014). Finding the missing link for big biomedical data. JAMA.

[CR75] Whitehead B, Andrews D, Shah A, Maidment G (2014). Assessing the environmental impact of data centres part 1: Background, energy use and metrics. Building and Environment.

[CR76] Wilbanks JT, Topol EJ (2016). Stop the privatization of health data. Nature.

[CR77] Williams E (2011). Environmental effects of information and communications technologies. Nature.

[CR78] Winner L, MacKenzie D, Wajcman J (1999). Do artifacts have politics?. The social shaping of technology.

